# Human Herpesvirus 6A Exhibits Restrictive Propagation with Limited Activation of the Protein Kinase R-eIF2α Stress Pathway

**DOI:** 10.1128/JVI.02120-16

**Published:** 2017-04-13

**Authors:** Eyal Sharon, Niza Frenkel

**Affiliations:** Department of Cell Research and Immunology and S. Daniel Abraham Institute for Molecular Virology, Tel Aviv University, Tel Aviv, Israel; University of California, Irvine

**Keywords:** ATF4, HHV-6, PKR, TCID_50_, eIF2α, viral replication

## Abstract

The eIF2α protein plays a critical role in the regulation of translation. The production of double-stranded RNA (dsRNA) during viral replication can activate protein kinase R (PKR), which phosphorylates eIF2α, leading to inhibition of the initial step of translation. Many viruses have evolved gene products targeting the PKR-eIF2a pathway, indicating its importance in antiviral defense. In the present study, we focused on alternations of PKR-eIF2a pathway during human herpesvirus 6A (HHV-6A) infection while monitoring viral gene expression and infectious viral yields. We have found increased phosphorylated PKR as well as phosphorylated eIF2α coincident with accumulation of the late gp82-105 viral protein. The level of total PKR was relatively constant, but it decreased by 144 h postinfection. The phosphorylation of eIF2a led to a moderate increase in activating transcription factor 4 (ATF4) accumulation, indicating moderate inhibition of protein translation during HHV-6A infection. The overexpression of PKR led to decreased viral propagation coincident with increased accumulation of phosphorylated PKR and phosphorylated eIF2a. Moreover, addition of a dominant negative PKR mutant resulted in a moderate increase in viral replication. HHV-6A exhibits relatively low efficiency of propagation of progeny virus secreted into the culture medium. This study suggests that the replicative strategy of HHV-6A involves a mild infection over a lengthy life cycle in culture, while preventing severe activation of the PKR-eIF2α pathway.

**IMPORTANCE** Human herpesvirus 6A (HHV-6A) and HHV-6B are common, widely prevalent viruses, causing from mild to severe disease. Our study focused on the PKR-eIF2α stress pathway, which limits viral replication. The HHV-6 genome carries multiple genes transcribed from the two strands, predicting accumulation of dsRNAs which can activate PKR and inhibition of protein synthesis. We report that HHV-6A induced the accumulation of phosphorylated PKR and phosphorylated eIF2α and a moderate increase of activating transcription factor 4 (ATF4), which is known to transcribe stress genes. Overexpression of PKR led to increased eIF2α phosphorylation and decreased viral replication, whereas overexpression of a dominant negative PKR mutant resulted in a moderate increase in viral replication. These results suggest that the HHV-6A replication strategy involves restricted activation of the PKR-eIF2α pathway, partial translation inhibition, and lower yields of infectious virus. In essence, HHV-6A limits its own replication due to the inability to bypass the eIF2α phosphorylation.

## INTRODUCTION

Human herpesvirus 6 (HHV-6) is a very widespread human pathogen with an estimated prevalence of nearly 100% of the world's population, commonly infecting children in the first 2 years of life (1, 2). HHV-6 was initially isolated from patients with lymphoproliferative disorders (3). The virus was found to be the causative agent of exanthem subitum (roseola infantum), a very prevalent disease of children that is characterized by a brief febrile infection with skin rash (4). Based on the association with roseola infantum and on the basis of DNA sequences and antigenicity, we and others have suggested that there are two distinct types of viruses: HHV-6A and HHV-6B (5, 6). HHV-6B is the main causative agent of roseola infantum. In a minority of children, HHV-6B infection causes grave disease with severe neurological complications, up to lethal encephalitis (7–9). HHV-6A and HHV-6B enter into a latency state, from which they can be reactivated (10–12). Reactivation of HHV-6B following immunosuppressive treatment for bone marrow and other transplantations can result in delayed transplant engraftment and central nervous system (CNS) complications, as well as lethal encephalitis (13–17). Thus far, there is no lytic disease associated with HHV-6A. Several studies have suggested linkage to autoimmune diseases, including multiple sclerosis (18, 19), connective tissue diseases (20), and Hashimoto's thyroiditis (21).

Viruses exhibit a wide range of mechanisms for subverting the host defense functions. In this paper we address the control of mRNA translation for successful HHV-6 replication. There are two main regulatory steps which control the polypeptide chain initiation: the activity of the initiator Met-tRNA-binding factor eIF2 and the formation of the eIF4F mRNA cap-binding complex (22). eIF2 is a G protein heterotrimer composed of three subunits, α, β, and γ (23). It is responsible for the delivery of the initiator methionine tRNA (Met-tRNAi^Met^) along with GTP to the small 40S ribosomal subunit, inducing the first step of translation initiation (24). Formation of active eIF2 (GTP bound) is necessary for formation of a stable ternary complex (eIF2·GTP·Met-tRNAi^Met^), promoting mRNA AUG codon recognition. During AUG selection by the ribosome, GTP is hydrolyzed by the GTPase-accelerating protein (GAP) activity and release of P_i_, reducing eIF2 affinity for Met-tRNAi and its release from the ribosome (25). To participate in a subsequent round of Met-tRNAi recruitment to the ribosome, eIF2 must be reactivated to the GTP-binding form. eIF2B is a guanine nucleotide exchange factor (GEF) for its GTP-binding protein partner eIF2 (26). Phosphorylation of the eIF2α subunit on Ser^51^ converts eIF2 from a substrate to an inhibitor of eIF2B and thereby decelerates cellular protein synthesis (27). The ratio of eIF2 to eIF2B is particularly important because phosphorylation of eIF2α can cause a stoichiometric inhibition of eIF2B by forming a stable GDP·eIF2 (eIF2αP)·eIF2B complex. Therefore, inhibition of protein synthesis occurs only when the molar extent of eIF2α phosphorylation exceeds the molar level of eIF2B. Measurements of eIF2 and eIF2B levels in diverse tissues have shown that the absolute level of eIF2 varies to a limited extent, as opposed to variable levels of eIF2/eIF2B ratios (between 2.5-fold and more than 10-fold) (28, 29). These findings indicate that the cells may differ significantly in their content of eIF2B and thus in their sensitivity to eIF2α phosphorylation.

Although the phosphorylation of eIF2α leads to downregulation of translation, the translation of several mRNAs containing short upstream open reading frames (upORFs) in their 5′ leader sequences increases following eIF2α phosphorylation. The activating transcription factor 4/5 (ATF4/5) and JunD mRNAs are regulated by this unique mechanism (30). The ATF4-coding region is located downstream of two short ORFs which are translated constantly in unstressed cells (31). Under stress conditions, phosphorylation of eIF2α, and the resulting reduction in the level of GTP-bound eIF2, the time required for the scanning ribosomes to become competent to reinitiate translation increases. The delayed reinitiation allows the ribosomes to scan through the inhibitory upORF2 and reinitiate at the ATF4-coding region (22, 31). ATF4 can directly or indirectly regulate expression of a large number of genes involved in metabolism and amino acid transport, the redox status of the cell, signaling, transcription, and apoptosis (32).

Phosphorylation of eIF2α on Ser^51^ is be carried out by a family of four serine-threonine kinases (EIF2AKs): the heme-regulated inhibitor kinase (HRI, EIF2AK1) (33, 34), the double-stranded RNA (dsRNA)-dependent protein kinase (PKR, EIF2AK2) (35–38), PERK (EIF2AK3) (39, 40), and GCN2 (EIF2AK4) (41). PKR is the only member of the eIF2a kinase family that is induced by interferon (IFN), accenting its part in the immune response. PKR is expressed constitutively but is not functional until activated. The classical activator of PKR is dsRNA which is cooperatively bound by tandem RNA-binding motifs (RBMs) at the N terminus of the PKR, leading to relief of steric inhibition of the kinase domain and colocalization of two PKR monomers forming a complex with dsRNA molecule. This allows back-to-back dimerization with the kinase domain facing outwards, leading to concomitant autophosphorylation of the critical threonine residues 446 and 451 in the activation loop which stabilizes the active dimeric enzyme (42, 43). PKR plays a major role in regulating eIF2α in response to virus infection. It is a key executor of this antiviral response, mediating an antiviral state in the infected cell and neighbor cells (44, 45).

Given the role of PKR, many viruses express proteins or RNAs that inhibit PKR or its downstream effects. The inhibitory mechanisms include sequestration of viral dsRNA by a viral protein and prevention of PKR activation through direct interaction with viral proteins or viral decoy RNA (46–49), regulation of eIF2α phosphorylation through a viral pseudosubstrate (50–52), regulation of eIF2α phosphorylation through recruitment of a cellular phosphatase (53), degradation of PKR (54–56), and relocalization of PKR (57).

Although HHV-6 is one of the most successful viral parasites, with a prevalence of nearly 100% in the world's population, the intracellular mechanisms employed by HHV-6 for immune evasion are thus far insufficiently characterized. It was shown that HHV-6 can negatively regulate IFN-β gene induction, utilizing IE1 protein. The inhibition of the IFN-β is a consequence of disturbance of IFN regulatory factor-3 (IRF3) dimerization, which reduced the activation of the IFN-β promoter (58). A vast amount of information has been accumulated with regard to manipulation of the PKR-eIF2α pathway by other human herpesviruses, including herpes simplex virus (HSV) (48, 53, 59, 60), Epstein-Barr virus (EBV) (49), cytomegalovirus (CMV) (61–63), and Kaposi's sarcoma-associated herpesvirus (KSHV) (47), but to the best of our knowledge no studies with regard to the effects of HHV-6 on this specific route have been reported. This paper describes the alterations in the PKR-eIF2α pathway during HHV-6A infection in parallel with the kinetics of viral replication.

## RESULTS

### Viral protein expression during HHV-6A infection.

**FIG 1:**
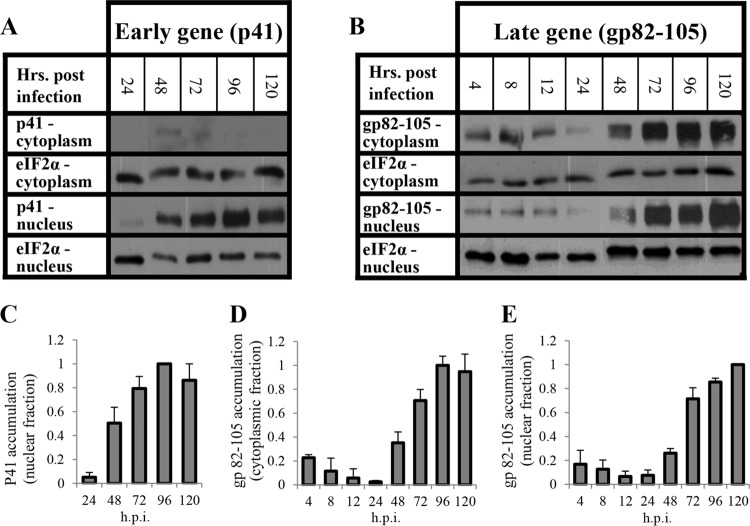
Accumulation of viral proteins during HHV-6A infection. SupT1 cells were infected with HHV-6A. The cells were harvested and fractionated into cytoplasmic and nuclear fractions at 4, 8, 12, 24, 48, 72, 96, or 120 hpi. The protein samples (15 μg/lane) were electrophoretically separated in a 10% SDS-polyacrylamide gel. (A) Western blots reacted with the P41 MAb (early gene) and the eIF2α antibody, serving as the housekeeping gene. (B) Blots reacted with the gp82-105 complex MAb (late gene) and the eIF2α antibody. (C) Graphic representation of the results relative to the highest level of P41 protein/eIF2α as detected in the nuclear fraction. (D and E) Graphic representation of the results relative to the highest level of gp82-105 complex/eIF2α as detected in the cytoplasmic and nuclear fractions. The results are shown as means with standard deviations based on four distinct experiments.

To follow the kinetics of viral replication, we monitored the accumulation of viral proteins in the cytoplasmic and nuclear fractions of infected SupT1 T cells. The cells were infected with HHV-6A with an input multiplicity of infection (MOI) of 2 50% tissue culture infective doses (TCID_50_)/cell. Protein samples were extracted at 4, 8, 12, 24, 48, 72, 96, and 120 h postinfection (hpi) and tested for the accumulation of the early (β) P41 DNA polymerase accessory protein, encoded by the early U27 gene (64), and the late (γ) gp82-105 complex, encoded by U100 (65). P41 was first detected at 24 hpi, and it increased accumulation at 48, 72, and 96 hpi (Fig. 1A and C). It has a nuclear localization signal, and, as expected, it was recovered solely in the nuclear fraction. These results also reflected an adequate separation of the nuclear and cytoplasmic fractions. Low levels of the HHV-6A gp82-105 complex were detected at 4, 8, 12, and 24 hpi in both the cytoplasmic and nuclear fractions, most likely representing proteins associated with the inoculated viral particles (Fig. 1B, D, and E). The level of the gp82-105 complex gradually decreased up to 24 hpi. *De novo* synthesis of the envelope complex started at 48 hpi and kept accumulating up to 120 hpi.

### HHV-6A infection leads to activation of the PKR-eIF2α pathway.

**FIG 2:**
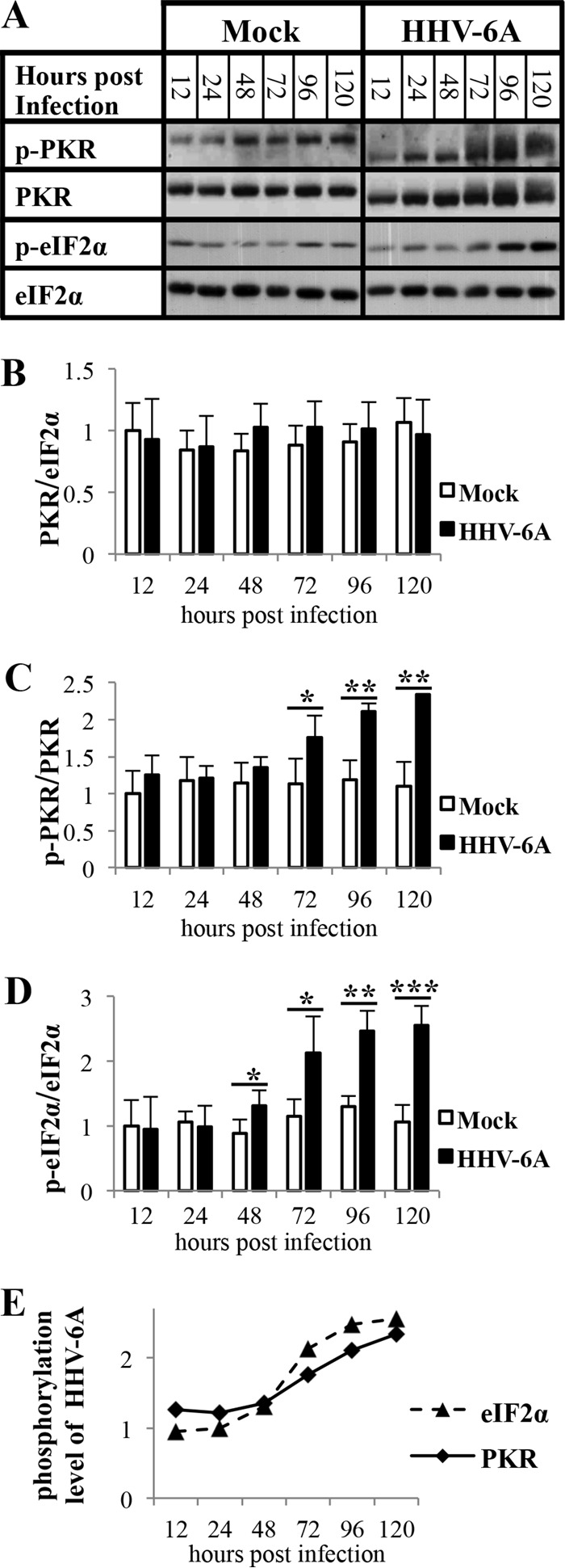
HHV-6A infection leads to PKR and eIF2α phosphorylation starting at 48 hpi. SupT1 cells were either mock infected or infected with HHV-6A. The cells were harvested at 12, 24, 48, 72, 96, and 120 hpi and proteins extracted from the cytoplasmic and nuclear fractions. (A) Western blots of mock-infected and virus-infected cytoplasmic lysates. The blots were tested with antibodies to PKR, PKR phosphorylated at residue T^451^, eIF2α phosphorylated at residue Ser^51^, and eIF2α as a housekeeping gene. (B to D) Graphic representation of the results relative to the mock level of PKR accumulation (PKR/eIF2α), phosphorylated PKR (p-PKR/PKR), and phosphorylated eIF2α protein (p-eIF2α/eIF2α). The intensities of PKR, phosphorylated PKR, and phosphorylated eIF2α were corrected for loading variations by the intensity of eIF2α. (E) Comparison between PKR and eIF2α phosphorylation (p-PKR/PKR and p-eIF2α/eIF2α). Results are shown as the means with standard deviations based on four distinct experiments. *, *P* < 0.05; **, *P* < 0.01; ***, *P* < 0.001 (compared with two samples).

We expect that upon infection there will be transcription of the two strands of viral DNA and increased accumulation of dsRNA, a classical activator of PKR. To follow the alterations of the PKR-eIF2α pathway along the infection, the cytoplasmic and nuclear protein extracts were quantified for PKR and eIF2α, employing antibodies to the N-terminal PKR and full-length eIF2α, serving as a housekeeping gene. The phosphorylation of PKR was followed by treatment with phosphospecific antibody to T^451^ and the phosphorylation of eIF2a by phosphospecific antibody to S^51^. These phosphorylation sites are critical for the activation of the proteins (27, 43). As shown in Fig. 2A and quantified in Fig. 2B, the level of total PKR was relatively constant in the cytoplasmic fractions of the uninfected and HHV-6A-infected cells. Furthermore, in the uninfected cells there was a steady, low phosphorylation of PKR (p-PKR/PKR) from 12 to 120 hpi (Fig. 2C). In contrast, in the infected cells, there was significant increase of PKR phosphorylation, over 2-fold, between 72 to 120 hpi. The increased phosphorylation was accompanied by a shift in molecular weight, characteristic of PKR phosphorylation (Fig. 2A). No such shift was found in the samples of the mock-infected cells. The phosphorylations of PKR and eIF2α increased coordinately (Fig. 2D and E) in the infected cells, showing a 2.5-fold increase of the phosphorylated eIF2α. The activation of the PKR-eIF2α pathway occurred in parallel with massive accumulation of the late viral protein gp82-105 at 72 hpi (Fig. 1B, D, and E).

### HHV-6A propagation at the late stage of infection.

**FIG 3:**
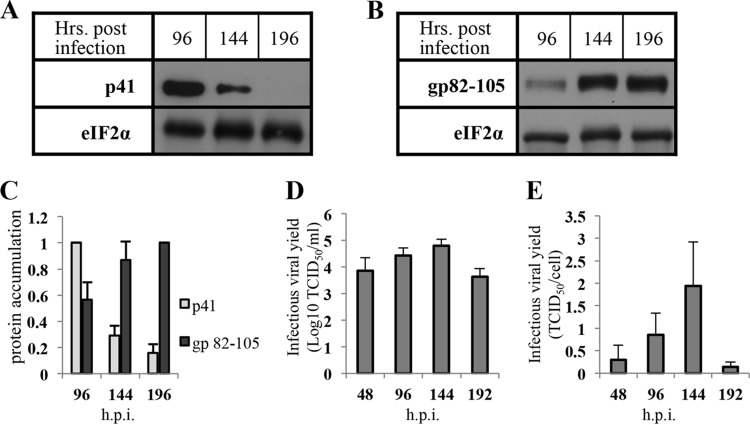
Accumulation of viral proteins and infectious viral yield in the late phase of HHV-6A infection. SupT1 T cells were treated as described for Fig. 5. Cell culture medium was harvested and frozen at 48, 96, 144, and 192 hpi. The media were later thawed and virus titer (TCID_50_) measured. (A and B) Western blots of viral protein. The membranes were reacted with antibodies to eIF2α, P41, and glycoprotein complex gp82-105. (C) Graphic representation of protein accumulation of P41/eIF2α and gp82-105 complex/eIF2α. (D) Graphic representation of viral yields (TCID_50_/ml) that were calculated from medium samples during the infection. (E) Graphic representation of viral yields versus infected cell number (TCID_50_/cell) values. Results are shown as means with standard deviations from three distinct experiments.

We further analyzed the kinetics of viral replication at the terminal stages of viral infection. Cell cultures were infected at an MOI of 2 TCID_50_/cell, and whole-cell proteins were prepared at 96, 144, and 192 hpi (Fig. 3A to C). P41 protein exhibited early (β gene) kinetics (Fig. 3A to C), with increased expression by 96 hpi followed by decreased accumulation at 144 hpi and 192 hpi The gp82-105 complex followed late (γ gene) kinetics, with increased expression between 96 and 192 hpi (66, 67).

**FIG 4:**
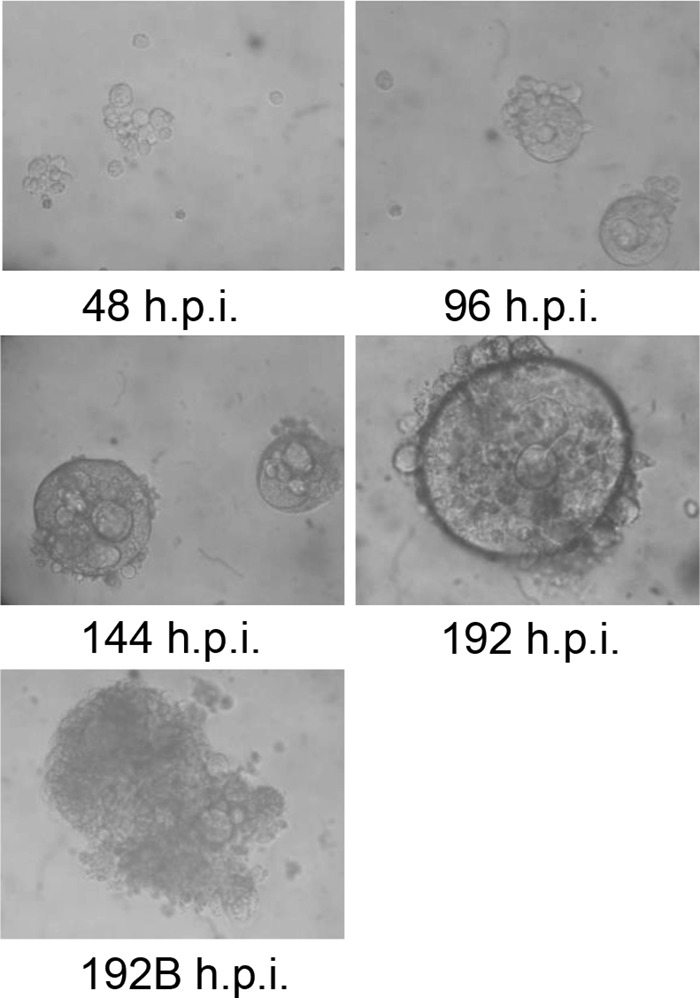
Viral CPE in late phase of HHV-6A infection. SupT1 T cells were infected at 2 TCID_50_/cell. The cell cultures were monitored at 48, 96, 144, and 192 hpi by light microscopy (magnification, × 200), and representative images were taken. 192B (lower panel), an image showing burst cells.

To quantify infectious viral yield (TCID_50_/ml) as well as infectious viral yield per cell (TCID_50_/cell), the medium was harvested at 48, 96, 144, and 192 hpi, and the titer of the secreted virus was determined. There was a 6-fold increase of secreted virus from 48 to 144 hpi, followed by 13-fold decrease between 144 and 192 hpi (Fig. 3D and E). The infectious viral yield at 192 hpi was very low. Moreover, infectious viral yields per cell showed moderate viral propagation, corresponding to the highest value of 2 TCID_50_/cell, similar to the input MOI (Fig. 3E). Representative images of infected cultures at different time points are shown in Fig. 4. CPE developed gradually across the infection from 48 hpi onward. At 96 hpi, the infected cultures contained large aggregates of small ballooning cells, which were enlarged by 144 hpi. At 192 hpi, approximately 50% of the ballooning cells burst and were destroyed.

### Low level of PKR protein at the termination of infection.

**FIG 5:**
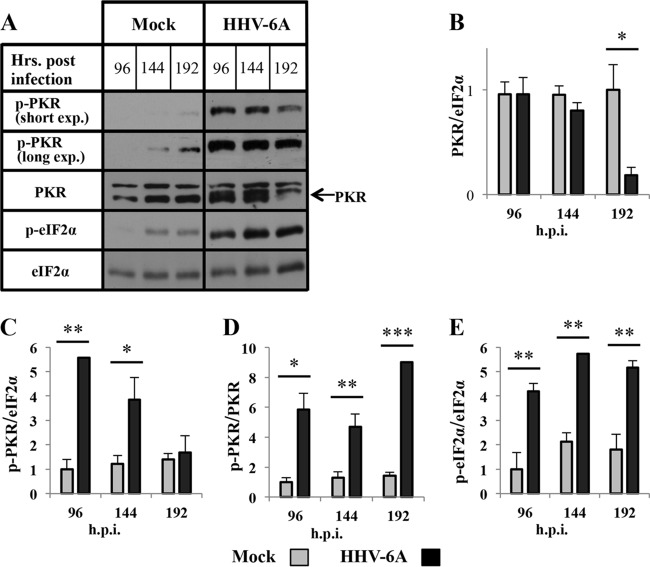
PKR and eIF2α hyperphosphorylation and PKR degradation in the late phase of HHV-6A infection. Cells cultures were either mock infected or infected with HHV-6A. The cells were harvested at 96, 144, and 196 hpi, and whole-cell proteins were prepared. (A) Western blots of mock-infected and virus-infected lysates. The membranes were reacted with antibodies to PKR, p-PKR, p-eIF2α, and eIF2α. (B to E) Graphic representation of the results relative to the mock level of PKR/eIF2α, p-PKR/PKR, p-PKR/eIF2α, and p-eIF2α/eIF2α. Results are shown as means with standard deviations based on three distinct experiments. *, *P* < 0.05; **, *P* < 0.01; ***, *P* < 0.001 (compared with two samples).

To follow the alternations of the PKR-eIF2α pathway at later stages of infection, whole-cell proteins were prepared at 96, 144, and 192 hpi. The blots were tested with antibodies to total PKR, p-PKR, eIF2α, and p-eIF2α. It is noteworthy that whole-cell extracts also contain the nuclear PKR and eIF2α, proteins which do not participate in translation regulation. In the infected cells, the level of total PKR decreased progressively from 144 to 192 hpi. By 192 hpi it was significantly reduced, exhibiting an approximately 5-fold lower level than in the mock-infected cultures (Fig. 5A and B). The accumulation of phosphorylated PKR (p-PKR/eIF2α) decreased 3-fold between 96 and 192 hpi (Fig. 5A and C). The low accumulation of phosphorylated PKR at 192 hpi reflected the reduced level of total PKR in the infected cultures. In the mock-infected cultures, there was a steady low background level of phosphorylated PKR from 96 to 192 hpi (Fig. 5C). PKR phosphorylation (p-PKR/PKR) in the infected cultures increased progressively and was approximately 5-fold higher than in the mock-infected cells (Fig. 5D). The phosphorylation level of eIF2α was about 2.5-fold higher in cells exposed to HHV-6A than in mock-infected cells (Fig. 5E). Moreover, there was no correlation between the phosphorylation level of the eIF2α and the accumulation of phosphorylated PKR molecules (p-PKR/eIF2α) at 192 hpi; whereas there was a significant decrease in the quantity of phosphorylated PKR, there was no parallel decrease in eIF2α phosphorylation.

### HHV-6A infection leads to a moderate increase of ATF4 protein.

**FIG 6:**
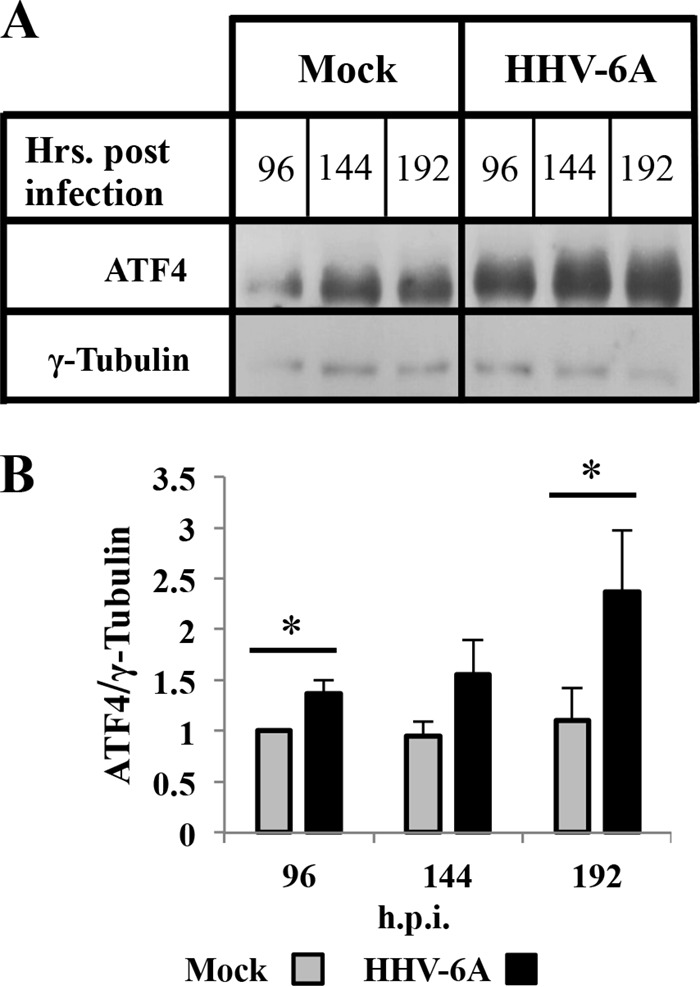
HHV-6A infection leads to a moderate increase in ATF4 accumulation. SupT1 T cells were treated as described for Fig. 5. (A) The blots were reacted with antibodies to ATF4 and γ-tubulin (loading control). (B) Graphic representation of ATF4/γ-tubulin protein accumulation ratio. The data are means with standard deviations based on three distinct experiments.

The phosphorylation of eIF2α reduced global translation, enabling the cell to respond effectively to stress conditions, including the induction of ATF4. Accumulation of phosphorylated eIF2α beyond the threshold level resulted in a significant decrease in the availability of active ternary complexes, global reduced translation, and the translation of ATF4. To follow the accumulation of ATF4 protein, blots of whole-cell proteins were reacted with antibodies to ATF4 and γ-tubulin, serving as a housekeeping gene. The results showed (Fig. 6) that in the infected cells there was a moderate increase of ATF4 protein at 96 and 144 hpi, reaching a 2-fold increase by 192 hpi. The increased eIF2α phosphorylation during infection between 96 and 192 hpi resulted in a moderate inhibition of protein synthesis, as was apparent by the increase of ATF4 expression (Fig. 5 and 6).

### Construction of amplicon-6 expression vectors carrying the PKR and a dominant negative PKR mutant.

To test the direct involvement of PKR in eIF2α phosphorylation as well as in viral replication, we produced amplicon-6 vectors with active PKR or a dominant negative PKR mutant. We employed the amplicon-6–green fluorescent protein (GFP) vector previously derived in our laboratory (68) to obtain efficient expression of the selected foreign gene(s) in lymphocytes. The vector contains the HHV-6A DNA replication origin, the composite packaging signals pac-1 and pac-2, and the selected transgene(s). Transfection of SupT1 cells with amplicon-6 vector in the presence of HHV-6A helper virus produces large concatemeric genomes with multiple reiterations of amplicon units, leading to high expression of the transgene(s). An amplicon-6–GFP–red fluorescent protein (RFP) vector was used as a control (Amp.control). We replaced the GFP gene with PKR containing an additional 81 bp (Amp.PKR), yielding a 71-kDa protein which allows it to be separated from the endogenous 68-kDa PKR. We also produced an amplicon carrying a dominant negative PKR mutant (Amp.ΔPKR), exhibiting a deletion of 6 amino acids (aa) at positions 361 to 366 of the PKR protein, using PCR-driven overlapping extension mutagenesis. These 6 amino acids were previously found to modulate the kinase activity toward inhibition of eIF2α phosphorylation and the formation of a hybrid wild-type PKR-ΔPKR dimer, resulting in an inactive kinase (69).

### PKR plays a major role in the phosphorylation of eIF2α during viral infection.

**FIG 7:**
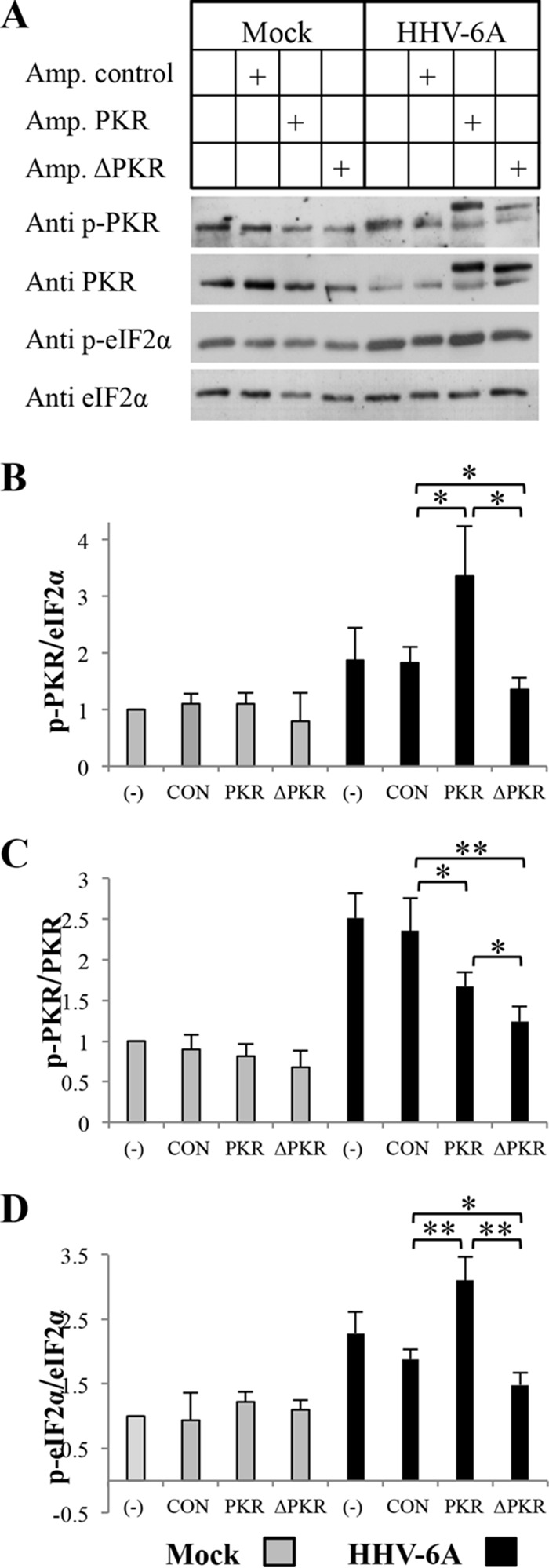
Increasing accumulation of phosphorylated PKR and eIF2α following overexpression of PKR. SupT1 T cells were untransfected (−) or transfected with amplicon-6–RFP–GFP (CON), amplicon-6–PKR–RFP (PKR), or amplicon-6–ΔPKR–RFP (ΔPKR). Twenty-four hours later, the transfected cells were mock infected or superinfected with HHV-6A. Protein samples were prepared at 96 hpi. (A) Western blots of the cytoplasmic fraction. The protein extracts were loaded on a 10% SDS-polyacrylamide gel (15 μg/lane) and then reacted with antibodies to PKR, phosphorylated PKR, phosphorylated eIF2α, and eIF2α. (B to D) Graphic representation of the results relative to the mock (−) level of p-eIF2α/eIF2α (B), p-PKR/PKR (C), and p-PKR/eIF2α (D). Results are shown as means with standard deviations based on four distinct experiments. *, *P* < 0.05; **, *P* < 0.01.

There are four members of the eIF2α kinase family. To test PKR involvement in eIF2α phosphorylation, parallel cultures of SupT1 T cells were untransfected or transfected with Amp.control, Amp.PKR, or Amp.ΔPKR. At 24 h posttransfection, the cells were mock infected or superinfected with HHV-6A. The cultures were monitored daily for development of cytopathic effect (CPE), employing fluorescence microscopy. The blots of the cytoplasmic proteins were extracted at 96 hpi and then were reacted with antibodies to PKR, p-PKR, eIF2α, and p-eIF2α. The results can be summarized as follows. (i) Whereas the cultures that received the control plasmid exhibited pronounced CPE with typical ballooning, the cultures transfected with active PKR manifested reduced CPE upon HHV-6A superinfection (data not shown). The cultures transfected with the dominant negative PKR expressed more pronounced ballooning CPE than the cultures receiving the control plasmid. (ii) As apparent in the Western blots (Fig. 7A) there were 3 sizes of PKR bands: an endogenous PKR protein of 68 kDa was expressed in uninfected and infected cells, an exogenous PKR protein of 71 kDa was expressed in the cells receiving the Amp.PKR with an added 81 bp, and an exogenous dominant negative PKR of 70 kDa was expressed the cells receiving the Amp.ΔPKR. In the infected cultures that received either the Amp.PKR or Amp.ΔPKR plasmid, there were high levels of exogenous PKR. In comparison to cultures that received the Amp.ΔPKR, the expression of the endogenous PKR was lower in cultures that received the Amp.PKR. (iii) In the mock-infected cells there were no significant alterations in the accumulation of phosphorylated PKR (p-PKR/eIF2α). In the cultures infected with 2 TCID_50_/cell without addition of plasmids (−) or upon addition of the Amp.Control, there was about a 1.8-fold increase in phosphorylated PKR. Significantly, in the cultures that received Amp.PKR there were 3.3-fold-higher levels of phosphorylated PKR, whereas in the cultures that received Amp.ΔPKR there was only 1.3-fold-increased phosphorylation compared to that in the mock-transfected samples (Fig. 7A and B). Moreover, the addition of the dominant negative PKR mutant protein led to reduced levels of phosphorylated endogenous PKR. (iv) In the mock-infected cultures there were no variations in the extent of phosphorylation (p-PKR/PKR) (Fig. 7A and C). However, infected cultures that were exposed to the exogenous active or dominant negative PKR showed lower levels of PKR phosphorylation than the transfection control and untransfected cultures. As expected, cultures transfected with the dominant negative PKR showed significantly lower phosphorylation than cultures that received the active PKR plasmid. It is apparent that the addition of exogenous PKR exceeded the phosphorylation capacity of the infected cells. Moreover, the exogenous dominant negative plasmid had very low phosphorylation compared to the endogenous band. (v) The phosphorylation of eIF2α was correlated with phosphorylated PKR, and it increased to the highest levels in the infected cultures receiving the active PKR plasmid (Fig. 7A and B). Moreover, the infected cultures transfected with the dominant negative PKR showed significantly lower phosphorylation of eIF2α than cultures receiving the active PKR, as well as the control vector. In summary, PKR was found to be a major kinase that phosphorylates eIF2α during the progression of HHV-6A infection.

### Overexpression of PKR inhibits viral replication.

**FIG 8:**
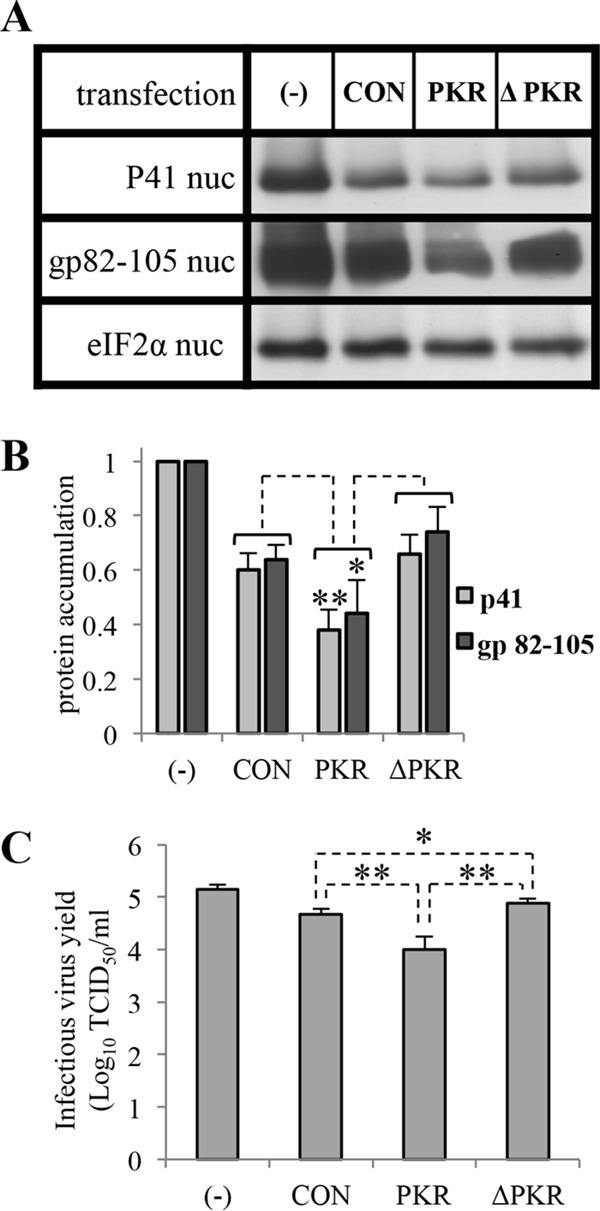
Decrease of viral protein synthesis and infectious virus yield following the overexpression of PKR. SupT1 T cells were treated as described for Fig. 7. (A) The blots of nuclear protein extracts were tested with antibodies to total eIF2α, P41 protein, and the glycoprotein complex gp82-105. (B) Graphic representation of viral protein accumulations corresponding to P41/total eIF2α and gp 82-105 complex/total eIF2α. (C) Graphic representation of infectious virus yield in medium samples that were harvested at 96 h postinfection. The results were normalized to the untransfected sample. Results are shown as means with standard deviations based on four distinct experiments.

To follow the effect of PKR overexpression on viral protein accumulation, the cultures were treated as discussed in the previous section, and protein samples from the nuclear fractions were tested for reactivity to monoclonal antibodies (MAbs) of HHV-6A p41 protein and gp82-105 and with eIF2α antibody (Fig. 8A and B). The results indicated low accumulation of these viral proteins in the culture that received the active PKR. Moreover, there was a moderate increase in protein accumulation in cultures that received the dominant negative PKR compared to the cultures that received the control vectors.

The yields of secreted infectious virus at 96 hpi (Fig. 8C) were 5-fold lower in cells transfected with Amp.PKR (1.02 × 10^4^/ml) than in the cultures transfected with control plasmid (4.70 × 10^4^/ml). Moreover, there was a moderate increase in infectious virus yield in cultures that received Amp.ΔPKR (7.79 × 10^4^/ml). The infectious virus yields in the untransfected cultures (1.37 × 10^5^/ml) were higher than the yields in the transfected cultures.

## DISCUSSION

Because the HHV-6 genome contains oppositely oriented promoters, the production of dsRNA is inevitable at late times postinfection, when the majority of viral genes have been transcribed. We have shown that viral infection leads to PKR activation simultaneously with the expression of the late gp82-105 protein complex. Increased PKR phosphorylation began at 72 hpi and continued until the destruction of the infected ballooning cells by 192 hpi.

The addition of exogenous PKR led to increased phosphorylated PKR, but the extent of phosphorylation (p-PKR/PKR) decreased, indicating that the added PKR exceeded the activation capacity of the viral dsRNA that was accumulated in the cells. Thus, a substantial fraction of the exogenous PKR molecules remained unphosphorylated. We suggest that the limited dsRNA accumulation results from restricted viral replication. Additional support for this hypothesis comes from the following findings. (i) In recent experiments we have found (data not shown) that there was a linear dependence between the viral input and progeny secreted infectious viral yields in cells infected with 1 to 10 TCID_50_/cell, representing low propagation efficiency. Moreover, when cells were infected with 1 to 10 TCID_50_/cell, similar ratios of PKR and eIF2α phosphorylation were detected at 96 hpi, suggesting that viral input, at this range, did not significantly alter the activation of the PKR-eIF2α pathway. When viral inputs exceeded 10 TCID_50_/cell, there was reduced accumulation of P41 early protein by 24 hpi, reduced infectious viral yield, and lower levels of phosphorylated PKR at 96 hpi. (ii) A recent study by Nukui et al. (70) identified an HHV-6A microRNA (miRNA) that inhibits the IE2 transactivation protein, encoded by the U86 gene. IE2 protein regulates viral lytic replication starting early postinfection. Mutation at the U86 miRNA recognition site dramatically increased expression of the three kinetic classes of viral mRNAs.

The phosphorylated eIF2α level was affected by the quantity of phosphorylated PKR. Whereas addition of PKR led to a substantial increase of eIF2α phosphorylation, the addition of the dominant negative PKR caused only a moderate decrease in phosphorylation. Furthermore, the overexpression of the dominant negative PKR mutant decreased the endogenous PKR phosphorylation, reflecting the competition on dsRNA binding. These results suggest that there is limited translation inhibition during HHV-6A infection, since the infection induces no upregulation of PKR, whereas an increased PKR level leads to a substantial inhibition of translation. As discussed above, HHV-6 can negatively regulate IFN-β gene induction (58), utilizing it to prevent massive accumulation of phosphorylated PKR. Moreover, our results verified that eIF2α phosphorylation during advanced stages of HHV-6A infection is caused by PKR.

The addition of exogenous PKR led to decreased viral protein accumulation as well as infectious virus yield, while addition of exogenous dominant negative PKR resulted in only a moderate increase in viral propagation. Therefore, we conclude that there is a negative correlation between eIF2α phosphorylation and HHV-6A propagation. In infected cells, without PKR upregulation, the accumulation of phosphorylated eIF2α barely goes over the threshold level necessary to inhibit translation. Based on our results, it is unlikely that HHV-6A utilizes a viral factor to inhibit PKR and eIF2α phosphorylation, apart from restrained production of dsRNA. Importantly, since diverse eIF2B levels are found in different cells/tissues (28), we suggest that the efficiency of HHV-6A lytic infection is strongly affected by the sensitivity of the particular cell/tissue to eIF2α phosphorylation. The induction of eIF2α phosphorylation by the virus might restrict its host range. Taking the findings together, the inability of HHV-6A to bypass the phosphorylation of PKR and eIF2α compels the virus to inhibit its own replication, enabling a moderate viral propagation even in the presence of a high level of PKR.

When monitoring PKR accumulation during the late phase of HHV-6A infection, a reduction was observed at 144 hpi, and the protein was barely detected at 192 hpi (Fig. 5A and B). The reduced PKR levels paralleled the destruction of the ballooning cells, indicating the endpoint of infection. Earlier studies have shown that viral proteins can induce the proteasomal degradation of PKR in order to escape antiviral cell response. Such interaction is exemplified by the NSs protein of Rift Valley fever virus (RVFV) (55, 56). PKR degradation was also reported for poliovirus (54). However, these studies found that PKR degradation occurred at an early stage of viral infection, enabling the escape from the host PKR-eIF2α pathway and efficient replication. Moreover, PKR protein is implicated in triggering cell death in response to viral infections. It has been shown that proteolytic cleavage of PKR by the apoptotic initiator caspase-8 and effectors caspase-3 and -7 liberates an active eIF2α kinase domain (71). In a preliminary experiment (data not shown), we found that both PKR and pro-caspase 3 were intact, suggesting that the reduced PKR was not caused by apoptosis. In summary, we assume that since the reduction of PKR levels started after the majority of progeny virions were secreted from the cells, PKR disappearance resulted from a cellular mechanism(s) preceding the destruction of ballooning cells.

We have shown that HHV-6A infection induces an increase in eIF2α phosphorylation (2.5-fold) coupled with a moderate increase (1.5-fold) in ATF4 protein accumulation by 144 hpi. These findings might suggest viral involvement in reducing translational arrest by interruption the binding of p-eIF2α to eIF2B. An alternative explanation for these finding could be the nature of the SupT1 cells processing relatively high levels of eIF2B, leading to improved resistance to eIF2α phosphorylation. Furthermore, we found a substantial increase in ATF4 accumulation following treatment with tunicamycin (data not shown), inducing the unfolded protein response accompanied by phosphorylation of PERK and eIF2α, suggesting that the infection induces only a moderate translation arrest.

There are only limited data available with regard to the role of ATF4 in herpesvirus infections. In HSV-1 infection, increased accumulation of ATF4 was detected at the final stage of the infection coincident with the completion of virion assembly and egress (72). Moreover, it was suggested that the induction of ATF4 and CHOP, an apoptotic factor downstream of ATF4, may trigger apoptosis, assisting release of newly synthesized viral particles from host cells. Human CMV (HCMV) and murine CMV (MCMV) activate the PERK signaling pathway and induce robust accumulation of ATF4 as well as a limited increase in phosphorylated eIF2α (73–75). Moreover, ATF4 promotes viral DNA synthesis and late gene expression. Interestingly, HCMV does not cause translation attenuation despite phosphorylation of eIF2α. In similarity with HSV-1, the final stages of HHV-6A infection (from 144 hpi onward) are accompanied by high ATF4 levels.

Consistent with microarray time-dependent cluster analysis of the herpesvirus lytic cascade, the HHV-6 genes can be clearly classified as immediate early (IE), early (E), and late (L) genes (76). We have focused on viral protein accumulations up to 8 days postinfection. We found that the P41 protein followed early kinetics. It was first detected at 24 hpi, peaked at 96 hpi, and was barely detected at 192 hpi. The P41 protein is an accessory for viral DNA polymerase, and the reduction of its accumulation at 144 hpi may indicate termination of new viral DNA replication. The late gp82-105 complex is a component of the mature viral particle. It was detected as early as 4 hpi and thereafter decreased up to 24 hpi, reflecting the entry and degradation of input virion structural proteins. *De novo* synthesis of the complex started at 48 hpi, and it kept accumulating up to 192 hpi. We followed the production of progeny virions by analyzing the accumulation of gp82-105 as well as virion maturation and secretion by measuring infectious virus yields, which approached the higher level by 144 hpi.

HHV-6A infection employing 2 TCID_50_/cell resulted in the secretion of approximately 2 TCID_50_/cell at 144 hpi. This was the highest level of infectious virus yield measured in our study. The total amount of the infectious progeny virions produced during the entire course of infection (from 48 to 192 hpi) could be estimated to be approximately 10-fold higher than the input virus (2 TCID_50_/cell). Additionally, based on our data it appears that virus secreted into the medium was unstable when kept in 37°C. To the best of our knowledge, there is no quantitative information available with regard to infectious virus produced in the course of infections of children with HHV-6A/6B. There are limited data available concerning viral propagation in culture. The available studies indicate only a moderate production of infectious progeny virions (77–79). In comparison to HHV-6A infection in SupT1 T cells, the propagation of other human herpesviruses, for example, HCMV (74, 80) and HSV-1 (81–83), exhibits higher efficiency in permissive cell cultures.

HHV-6 is one of the most successful viral parasites, with a prevalence of nearly 100% in the world's population. As discussed here, in comparison with other herpesviruses, HHV-6A is a slowly replicating virus, exhibiting moderate propagation in culture accompanied by relatively modest accumulation of viral dsRNA. We hypothesize that the HHV-6A replication strategy leads to restricted activation of the PKR-eIF2α pathway, enabling the completion of viral replication even without the utilization of viral elements to restrain this major translation arrest pathway.

## MATERIALS AND METHODS

### Cells and viruses.

SupT1 CD4^+^ human T cells were obtained from the NIH AIDS Research Respiratory. The SupT1 cells were cultured in RPMI 1640, 10% fetal bovine serum (FCS), and gentamicin (25 μg/ml). HHV-6A (U1102) was obtained from Robert Honess (National Institute for Medical Research, UK). Virus stocks were prepared from virus secreted into the medium and concentrated by centrifugation at 21,000 rpm for 2.15 h (4°C). Titers were determined using the endpoint assay, estimating the tissue culture infectious dosage for infecting 50% of the cells per ml (TCID_50_/ml).

### Transfection.

Cells (2 × 10^6^) were transfected with 13 μg of the test plasmid, employing a MP1000 microporator (Biodigital). The efficiency of transfection was determined by RFP expression.

### Plasmid construction.

The PKR gene was amplified from a brain cDNA library by PCR, employing the forward primer 5′-ATACCGGTCGCCACCATGGCTGGTGATCTTTCAGCAGG-3′ and the reverse primer 5′-TTACATTGATCATAACATGTGTGTCGTTCATTTTTCTCTG-3′. The PCR product was cloned into the amplicon-6–GFP vector (pNF1194), previously derived in our laboratory (68), containing the GFP gene driven by the HCMV promoter. The GFP gene was exchanged for the enlarged PKR ORF, encoding a 71-kDa protein (utilizing a deletion of one nucleotide in the stop codon, which adds 27 aa to its length). The RFP gene with the HCMV promoter was then introduced into the polylinker, employing the forward primer 5′-GAACCGGTGTCGACCCTGCAGCCCTAATAGTAATCA-3′ and the reverse primer 5′-GAACCGGTCTCGAGCGCTTACAATTTACGCGTTAAG-3′, generating the amplicon-6–PKR (pNF1511). The vector amplicon-6–ΔPKR (pNF1512) was constructed to have a dominant negative PKR mutant, which was made by deletion of a six-amino-acid sequence (positions 361 to 366), employing PCR-driven overlap extension mutagenesis. In addition to external primers used for PKR amplification, two primers were utilized for the mutagenesis: deletion forward primer 5′-GACTAAGTGCTTCTGTGATAAAGGGACCTTGGAACAATGG-3′ and deletion reverse primer 5′-GGTCCCTTTATCACAGAAGCACTTAGTCTTTGACCTTGAACTATTTTTGC-3′. Finally, the pNF1194 vector was utilized to produce the amplicon-6–control (pNF1510) by inserting the RFP gene similarly to amplicon-6–PKR construction.

### Antibodies.

Rabbit polyclonal antibodies to PKR (N-18), eIF2α (FL-315), and γ-tubulin (H-300) were purchased from Santa Cruz Biotechnology. Rabbit polyclonal antibodies to pPKRT451 (44-668) and peIF2αS51 (44-728) were purchased from Biosource International. Rabbit polyclonal antibody to ATF4 (ab-23760) was purchased from Abcam. Rabbit polyclonal antibodies to GFP (598) and RFP (PM005) were purchased from MBL. Mouse MAb 9A5, reactive to the HHV-6 P41 processivity factor encoded by the U27 gene, and mouse MAb 2D6, reactive to HHV-6A envelope glycoprotein complex gp82-105, were kind gifts from N. Balachandran. Horseradish peroxidase-conjugated secondary antibody (goat anti-rabbit 111-035-144 and goat anti-mouse 115-035-146) were purchased from Jackson ImmunoResearch Laboratories.

### Western blotting.

Whole-cell protein extracts were prepared by adding 100 μl of lysis buffer (including protease and phosphatase inhibitors) (50 mM Tris [pH 7.5], 280 mM NaCl, 0.5% NP-40, 0.2 mM EDTA, 2 mM EGTA, 1 mM dithiothreitol [DDT], 10 mM NaF, 10 mM β-glycerophosphate, 0.1 mM sodium orthovanadate, and 1 mM phenylmethylsulfonyl fluoride [PMSF] in isopropanol and 1:20 protease inhibitor cocktail [Roche]) to 2 × 10^6^ cells for 25 min at 4°C. The supernatant samples were frozen at −80°C. For preparation of cytoplasmic and nuclear extracts, the samples were first incubated in ice for 10 min with cytoplasmic lysis buffer (including protease and phosphatase inhibitors) (10 mM HEPES [pH 7.5], 10 mM KCl, 3 mM MgCl_2_, 0.05% NP-40, 1 mM EDTA, 1 mM DTT, 10 mM NaF, 10 mM β-glycerophosphate, 0.1 mM sodium orthovanadate, and 1 mM PMSF in isopropanol and 1/20 protease inhibitor cocktail) and then centrifuged at 400 × *g* for 5 min. The supernatant fluids (cytoplasmic extracts) were transferred into new cold tubes and were frozen at −80°C. The nuclear pellets were rinsed twice in cytoplasmic lysis buffer to further remove the cytoplasmic proteins from the nuclear fraction and then lysed with nuclear lysis buffer (50 mM HEPES [pH 7.9], 250 mM KCl, 1% NP-40, 5% glycerol, 0.1 mM EDTA, 1 mM DTT, 10 mM NaF, 10 mM β-glycerophosphate, 0.1 mM sodium orthovanadate, and 1 mM PMSF in isopropanol and 1/20 protease inhibitor cocktail). The samples were frozen and thawed three times and incubated on ice for 30 min. The insoluble material was pelleted in an Eppendorf centrifuge at 14,000 rpm for 7 min at 4°C. The supernatant fraction containing the nuclear extract was frozen at −80°C. After determining the concentration for each sample (Bio-Rad Dc protein assay), 10- to 50-μg protein samples were loaded on 10% SDS-polyacrylamide gels and transferred to nitrocellulose membranes (Whatman Protran, 0.45 μm) for Western blotting. The blots were blocked with 5% bovine serum albumin (BSA) in Tween 20–Tris-buffered saline (TTBS) for 1 h at room temperature and then were incubated for 2 to 24 h in 4°C with primary antibody (diluted 1:200 to 1:1,000 in TTBS containing 3% BSA). After being rinsed with TTBS, they were incubated with secondary antibody (diluted 1:5,000 to 1:50,000 according to the primary antibody in TTBS with 5% skim milk) for 60 min at room temperature and washed 4 times for 5 min each with TTBS. Finally, ECL mixture was added (Thermo SuperSignal West Pico chemiluminescent or PerkinElmer Lightning Ultra) for 3 min of incubation. The membranes were exposed to Fuji medical X-ray film, and the resulting protein bands were analyzed with a densitometer apparatus using the Microtek Bio-5000 scanner and the programs Scan wizard Bio and Image Quant TL. The antibodies were stripped from the nitrocellulose membranes using Restore stripping buffer (Pierce) accordingly to the manufacturer's protocol.
